# Categorization of echocardiograms by humans and pigeons

**DOI:** 10.3389/fpsyg.2025.1680346

**Published:** 2026-01-09

**Authors:** Odysseus R. P. Orr, Victor M. Navarro, Edward A. Wasserman, David Ouyang

**Affiliations:** 1 Department of Psychological and Brain Sciences, University of Iowa, Iowa, IA, United States; 2 School of Psychology, Cardiff University, Cardiff, Wales, United Kingdom; 3 Kaiser Permanente Northern California, Oakland, CA, United States

**Keywords:** category learning, comparative cognition, medical perception, two-alternative forced choice, visual aids

## Abstract

Categorizing medical samples is a difficult and time-consuming task that directly impacts patient outcomes. Recent technological advancements may hold the key to improving medical professionals' diagnostic accuracy. One of these advancements is EchoNet-Dynamic, a convolutional neural network that segments echocardiograms—ultrasound videos of the heart—producing a red overlay onto the left ventricle, the area of the heart relevant to diagnosis. We investigated the potential for EchoNet-Dynamic's segmentation to aid naïve non-clinician humans and pigeons in their diagnosis of cardiac function. Humans were trained to categorize either segmented or non-segmented echocardiograms as depicting normal or abnormal heart function. Then, roughly half of the subjects in each group were tested with videos of the opposite type they were trained with. We found that more humans trained with segmented videos adequately learned the task than those trained with non-segmented videos; they also learned more quickly, exhibited higher accuracies at the end of training, and reliably generalized to non-segmented videos during testing. Despite these apparent benefits, there was no general improvement in the accuracy of humans trained with non-segmented videos when testing with segmented videos. Pigeons, trained with segmented videos, successfully learned the task. However, unlike humans, they failed to generalize their learning to non-segmented videos, even after a fading procedure was employed. We conclude that EchoNet-Dynamic's segmentation is an effective visual aid that enhances learning and enables reliable transfer to non-segmented videos for humans, and provides a means of learning what otherwise might have been an incredibly difficult task for pigeons.

## 1 Introduction

Categorizing medical samples is a difficult and time-consuming task that can mean life or death for a patient. Despite extensive training, medical professionals' diagnoses are influenced by numerous factors. In the field of cardiology, for instance, the variability of cardiologists' assessments of heart function heavily depends on the imaging method used (Malm et al., 2004; Pellikka et al., 2018; Pillai et al., 2024). In extreme cases, different imaging methods can result in a lack of agreement among diagnosticians and, consequently, differing rates of misdiagnosis (Huang et al., 2017). Image quality also plays a crucial role in the reliability of diagnoses, as lower quality images can lead to less agreement among diagnosticians (Cole et al., 2015). This issue is particularly problematic when considering that differences in image quality often go undetected by individual diagnosticians (Cole et al., 2015). Another challenge arises when patients must rely on more generalist medical professionals to make specialized diagnoses. For example, in breast cancer assessments, non-specialists in breast histology show less diagnostic reproducibility compared to specialists and they tend to evaluate the severity of tumors less conservatively (Dunne and Going, 2001; Zhang et al., 2010).

Given the numerous challenges that medical professionals face, it is crucial to both standardize and improve diagnostic techniques (Lang et al., 2015). Many medical imaging tasks entail significant clinical heterogeneity, reflecting differences in opinion across both providers and the inherent subjectivity of certain diagnoses and assessments. To alleviate these issues, some have proposed replacing human diagnoses with diagnoses made by convolutional neural networks (CNNs). CNNs have demonstrated the ability to accurately predict patients' risk of disease (Betancur et al., 2018; Gandomkar et al., 2019) and even to perform diagnoses directly from medical samples (Ouyang et al., 2020; Zhang et al., 2018). Although CNNs demonstrate diagnostic strengths comparable to those of medical professionals, they are constrained by their computational requirements and reliance on available datasets, which can be difficult to obtain in large, high-quality, and well-labeled forms (Varoquaux and Cheplygina, 2022). Other research efforts suggest a more collaborative relationship between automated systems and medical professionals, aiming to develop tools that assist professionals throughout the diagnostic workflow. Recent research has developed methods to guide medical professionals' attention to the most diagnostic features of medical samples (Liao et al., 2022), enhance the images of the samples themselves (Firoz et al., 2016), and combine professional diagnoses with machine learning analysis (Gandomkar et al., 2019).

In addition to technological advancements, research has explored the use of surrogate animal models to study medical image perception. These studies represent a parallel approach to improving diagnostic accuracy by understanding the underlying perceptual and cognitive mechanisms, particularly with species that are affordable to house, extensively trainable, and whose prior experience is readily accounted for. Pigeons, for example, have shown remarkable abilities in domains such as histology, radiology and cardiology. In work done by Levenson et al. (2015), pigeons could accurately discriminate between benign and malignant tumors in histology samples and identify micro calcifications in mammograms. In a separate study, Navarro et al. (2020) demonstrated that pigeons could also reliably distinguish between normal and abnormal heart perfusion levels in humans. Although prior research has proven pigeons' competency as a model for studying medical image perception, their perception of medical videos remains unexplored. Pigeons have been shown to reliably discriminate and categorize actions (Asen and Cook, 2012), biological motion (Dittrich et al., 1998; Johansson, 1973; Giese and Poggio, 2003), and motion in general (Cook and Murphy, 2012; Dittrich and Lea, 1993). This evidence prompts us to ask whether pigeons can categorize medical stimuli where motion is a key factor.

**Figure 1:**
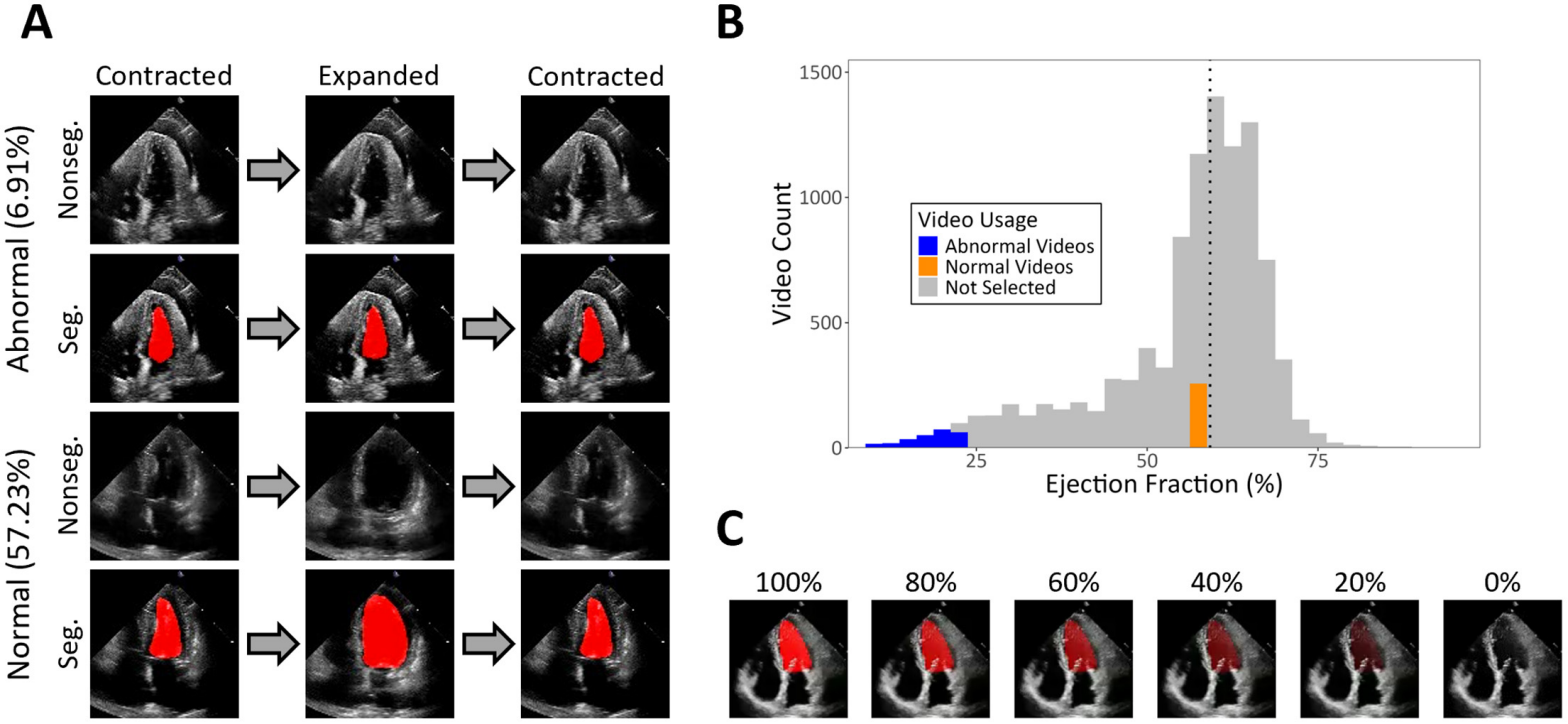
Exemplars that were shown during the experiment. **(A)** An exemplar from the abnormal category (top two rows) and an exemplar from the normal category (bottom two rows). The EF of each exemplar is depicted in parentheses. The first and third columns of this panel represent the most contracted frame of the exemplars. The second column represents the most expanded frame of the exemplars. The segmented versions of the exemplars can be seen below their non-segmented counterparts. **(B)** The distribution of EFs for the EchoNet-Dynamic dataset. The dotted line represents the median EF value of the dataset (59.21%). In blue are the abnormal videos we selected, in orange are the normal videos we selected, and in gray are the videos we did not select. **(C)** An exemplar with segmentations at various opacities by 20% increments.

Here, we evaluate the utility of a CNN, EchoNet-Dynamic (Ouyang et al., 2020), as an assistive tool for naïve non-clinician humans and pigeons diagnosing videos of cardiac function. EchoNet-Dynamic is a CNN trained to diagnose heart function using echocardiogram data by attending to, among other things, the relationship between the diastolic (relaxed) and systolic (contracted) volumes of the heart. EchoNet-Dynamic quantifies this relationship by utilizing self-made segmentations, or tracings, of echocardiograms to calculate the heart's left ventricular ejection fraction (EF). Abnormal hearts tend not to contract very much, leading to minimal movement and a lower EF, whereas normal hearts contract more, leading to greater movement and a higher EF. While the diagnostic power of EchoNet-Dynamic is itself impressive, its potential as a visual aid is made most apparent from its ability to create segmentations. Figure 1A depicts video frames from echocardiograms of an abnormal and normal heart, both with and without the segmentation of EchoNet-Dynamic. The added saliency that the segmentations provide could be a key tool in learning to diagnose heart function.

To evaluate the segmentation's utility for humans, we trained undergraduates at the University of Iowa to categorize echocardiograms as depicting normal or abnormal heart function. We were particularly interested in whether including the segmentation would enhance acquisition, so we trained subjects with either segmented videos or non-segmented videos. After training, we assessed whether subjects trained with segmented videos could generalize their learning to non-segmented videos and whether the accuracy of subjects trained with non-segmented videos would improve when tested with segmented videos. We found that more subjects trained with segmented videos learned the task than those trained with non-segmented videos; they also learned the task more quickly, achieved higher diagnostic accuracy by the end of training, and could reliably generalize to non-segmented videos. Despite this, there was no general improvement in the accuracy of those trained with non-segmented videos when testing with segmented videos. Pigeons, which we anticipated would have more difficulty with the task, were trained with segmented videos only. We were primarily interested in whether pigeons were capable of learning this difficult task, and, if so, whether the utility of EchoNet-Dynamic's segmentation was different for them than humans. We found that pigeons, like humans, could learn to accurately categorize segmented videos. Unlike humans, though, pigeons could not reliably generalize to even familiar videos without the segmentation. Even when we attempted to aid generalization by progressively fading the segmentation, none of the pigeons achieved adequate performance without it. We conclude that EchoNet-Dynamic's segmentation is an effective visual aid. For humans, it enhances learning outcomes and supports generalization to non-segmented videos. For pigeons, it provides a potentially necessary tool to learn the task.

## 2 Materials and methods

### 2.1 Humans

#### 2.1.1 Subjects

A total of 125 undergraduate students participated in the experiment. Sixty of them only underwent training with either segmented (*n* = 30) or non-segmented (*n* = 30) videos only, whereas 65 underwent both training and testing (*n* = 32 for those trained with segmented videos and tested with non-segmented videos; *n* = 33 for those trained with non-segmented videos and tested with segmented videos). We combined the training data of all subjects trained with segmented videos into one Segmented Training Group (*n* = 62), regardless of whether they were also tested with non-segmented videos. Similarly, we combined the training data of all subjects trained with non-segmented videos into one Non-segmented Training Group (*n* = 63). This seemed reasonable, as their training procedures were identical.

Subjects were recruited from the elementary psychology participant pool in the Department of Psychological and Brain Sciences at The University of Iowa. All subjects provided their informed consent prior to the start of the experiment and received course credit for their participation. All experimental procedures accorded with the Declaration of Helsinki and were approved by the Institutional Review Board at The University of Iowa (ID: 201808798; Approved: 08/22/2018).

#### 2.1.2 Apparatus

All human studies were carried out in an online modality, with subjects completing the task on their own personal computers.

#### 2.1.3 Stimuli

The full set of echocardiogram videos consisted of 10,030 left ventricle videos from the EchoNet-Dynamic database (Ouyang et al., 2020). The videos were either segmented, containing an EchoNet-Dynamic-generated red overlay that tracked the volume of the ventricle, or non-segmented, containing no overlay (see Figure 1A). EchoNet-Dynamic's segmentations were previously reported to be highly similar to those of expert human clinicians (Ouyang et al., 2020), so we saw no need for further quality control of segmented videos. Videos were 112 × 112 pixels in size and had an average duration of 3.46 s. Because subjects completed the task on their personal computers, the final size of the stimuli could not be controlled. Instead, the stimulus region was scaled so it occupied 500 × 500 pixels on the screen.

A total of 512 videos (256 per category) from the full set were selected to be used for both training and testing. For the abnormal category, we selected the 256 videos with the lowest EF. For the normal category, we attempted to select the first 256 videos above the median EF of the full dataset. Due to an experimenter error, however, the videos we selected fell slightly below the median. Due to the small magnitude of this discrepancy as well as the wide separability between the EFs of the categories we curated, we do not believe this error had any appreciable impact on our results. The distribution of EFs for the full dataset and our sample can be seen in Figure 1B.

Training videos were randomly sampled from the predefined set on a subject-by-subject basis without replacement (i.e., no videos repeated). Testing videos were randomly sampled in the same manner, but from videos of the opposite modality (i.e., if trained with segmented videos, testing included non-segmented videos, and vice versa). Because testing videos were sampled from the same predefined set of videos that were sampled from during training, testing included both familiar and novel videos of the opposite modality (e.g., if trained with segmented videos, testing included non-segmented versions of videos shown during training and completely novel non-segmented videos).

#### 2.1.4 Procedure

##### 2.1.4.1 Overview

Upon accepting the task, subjects were given a hyperlink to the online task, programmed using the jsPsych library (de Leeuw, 2015). Once on the website, they were welcomed with a consent form explaining the nature of the task, their compensation, and their rights as subjects. Once subjects consented to participate in the study, they were presented with task instructions (Supplementary material 1) and then quizzed via three, multiple-option questions (Supplementary material 2, following Le Pelley et al., 2019). Subjects started the task after correctly answering those three questions; any incorrect answers had them reread the instructions and try the quiz again. Upon completing the task, subjects were debriefed on the aims of the study. At no point were subjects explicitly instructed on the features relevant to diagnosis.

##### 2.1.4.2 Training with segmented or non-segmented videos

All subjects completed 300 training trials with segmented or non-segmented videos only. On each trial, a randomly sampled video began playing. After 2 s, a prompt [“Normal (F) or Dysfunctional (J)?” choice keys, counterbalanced) indicated to subjects that they were allowed to respond. Videos looped until subjects pressed one of the keys, after which visual feedback was given for 1 s (“Correct!” or “Error!” for correct and incorrect responses, respectively). Incorrect responses led to correction trials with the same video until a correct response was made. Data from correction trials were excluded from all statistical analyses.

##### 2.1.4.2 Testing with videos of the opposite modality seen in training

Subjects who underwent testing completed an additional 40 testing trials after training. Testing trials were identical to training, except that no feedback or correction trials were given.

#### 2.1.5 Analyses

##### 2.1.5.1 Overview

All data were subjected to logistic mixed-effects modeling with accuracy as the dependent variable and with all possible interactions among fixed factors included. Models were fit using the maximal random-effects structure supported by the data (Matuschek et al., 2017). All analyses were conducted in R, v4.4.2 (R Core Team, 2024) using the lme4 v1.1-35.5 (Bates et al., 2015), tidyverse v2.0.0 (Wickham et al., 2019), and emmeans v1.10.5 (Lenth, 2024) packages. The original data and code used for the analyses are available in the OSF repository at https://doi.org/10.17605/OSF.IO/7C9HK.

##### 2.1.5.2 Assessing acquisition with segmented vs. non-segmented videos

Training was divided evenly into six 50-trial blocks. Successful acquisition was defined as achieving a proportion of correct responses of at least 0.60 in the last block for both categories, which is above chance (0.50). Because no empirically established criterion exists for this task, we adopted this threshold to balance evidence of learning with humans' task constraints: they received only 300 trials with non-repeating stimuli, and they were given no instructions on what features to pay attention to.

To assess whether segmentation enhanced learning, we first conducted a two-proportion z-test to see if a greater proportion of subjects in the Segmented Training Group met criterion than those in the Non-segmented Training Group. Then, we used a logistic mixed-effects model to assess (1) whether the rate of acquisition was higher for the Segmented Training Group and (2) whether accuracy in the final block was higher for the Segmented Training Group. To account for non-linear learning rates and for potential differences in performance across categories, the model included the logarithm of 50-trial blocks (1 to 6, centered on the last block) and category (contrast coded with normal at 0.5 and abnormal at −0.5) as fixed factors. The model also included our primary factor of interest: group (contrast coded with segmented training at 0.5 and non-segmented training at −0.5). Finally, the model included random subject intercepts and random subject slopes for category and block. To focus on the dynamics of successful acquisition, only subjects who met criterion were included in this analysis.

##### 2.1.5.3 Assessing generalization from segmented to non-segmented videos

Our primary question in this phase was whether subjects from the Segmented Training Group could generalize their learning to non-segmented videos. We assessed this for non-segmented versions of the videos seen during training and for non-segmented videos that were completely novel.

Recall that 32 of the 62 subjects in the Segmented Training Group underwent testing; the remaining 30 only completed training. Only tested subjects who met the training criterion were included in this analysis. To assess generalization for these subjects, we compared performance on the 40 non-segmented testing trials with performance in the last block of training. We chose these training trials because they represent baseline performance at the end of training, and because they ensure a comparable sample size (50 trials) to compare testing trials to.

The model used to assess these data included video type (segmented training, familiar non-segmented testing, or novel non-segmented testing) as a fixed factor and random subject intercepts. Video type was dummy coded to assess two comparisons: one between segmented training and familiar non-segmented testing, and one between segmented training and novel non-segmented testing. To maximize power for our primary question, we excluded category as a fixed factor in this analysis; category-level accuracies are reported in Supplementary material.

##### 2.1.5.4 Assessing generalization from non-segmented to segmented videos

Our primary question in this phase was whether subjects from the Non-segmented Training Group would benefit from EchoNet-Dynamic's segmentation. However, a related question arises when considering this: Does the segmentation differentially benefit those who failed to meet the training criterion? To address this, we included both subjects who met the criterion and those who did not in our analysis. As in our analysis of the Segmented Training Group, we assessed this for segmented versions of the videos seen during training and for segmented videos that were completely novel.

Recall that 33 of the 63 subjects in the Non-segmented Training Group underwent testing; the remaining 30 only completed training. Only subjects who underwent testing were included in this analysis. Like before, we compared performance on the 40 segmented testing trials with performance on non-segmented videos in the last 50-trial block of training. The model used to assess these data included video type (non-segmented training, familiar segmented testing, or novel segmented testing) and learner (contrast coded with learner at 0.5 and non-learner at −0.5) as fixed factors. The model also included random subject intercepts. Video type was dummy coded to assess two comparisons: one between non-segmented training and familiar segmented testing, and one between non-segmented training and novel segmented testing. To maximize power, we excluded category as a fixed factor in this analysis; category-level accuracies are reported in Supplementary Material.

### 2.2 Pigeons

#### 2.2.1 Subjects

Four pigeons (*Columba livia*) participated in the experiment. They were food-deprived to 85% of their free-feeding weight and given free access to water and grit. All four pigeons had previously participated in visual categorization experiments, forgoing the need for additional training to respond in the operant conditioning chambers. However, the birds had only been exposed to still images, requiring a brief pre training phase to accustom them to the videos. The housing and training procedures were approved by the Institutional Animal Care and Use Committee at the University of Iowa (ID: 9021693; Approved: 02/13/2019).

#### 2.2.2 Apparatus

**Figure 2:**
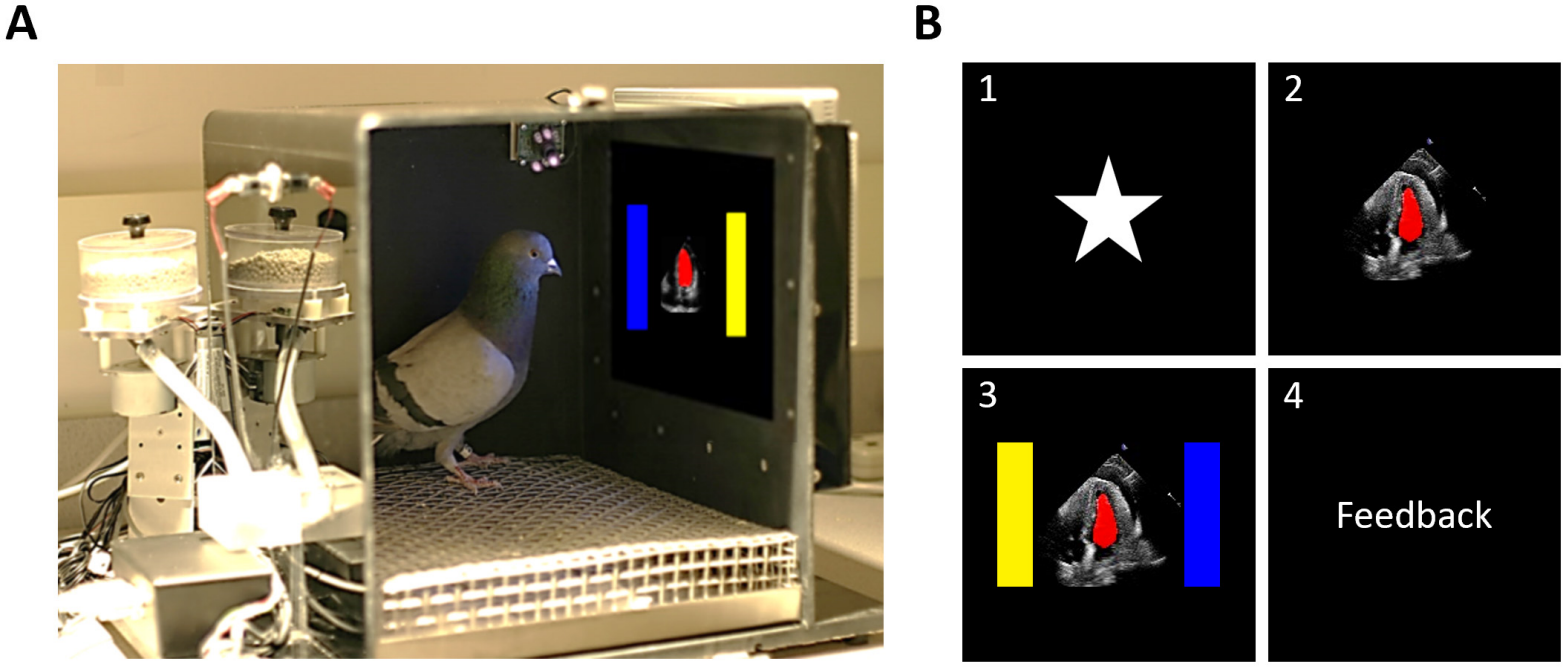
Apparatus and trial structure for the pigeons. **(A)** One of the operant conditioning chambers in which the pigeons performed. Stimulus sizes and placements are approximate. **(B)** The order of events for a typical trial. The number labels are presented as a visual aid only and were not present during the experiment. The panel labeled “4” was not present during the experiment either; it merely indicates when feedback (food reward/no reward) occurred on a given trial. Stimulus sizes and placements are approximate.

We used four 36 × 36 × 41 cm conditioning chambers (detailed in Gibson et al., 2004), located in a dark room with continuous white noise. Figure 2A depicts one of the conditioning chambers. Each chamber was equipped with a 15-in LCD monitor (1,024 × 768 resolution) behind a resistive touchscreen. The visible portion of the screen was 28.5 × 17.0 cm. The screen had one 2.0 × 2.0 cm area for the start stimulus, a 6.0 × 6.0 cm area for the target stimulus, and two 4.5 × 4.5 cm areas for the choice buttons. The start stimulus and target stimulus were located 13.0 cm and 9.0 cm, respectively, above the wire mesh floor and were centered horizontally. Choice buttons were presented to the left and right of the target stimulus, with 3.5 cm of separation. A rotary dispenser delivered 45-mg food pellets through a vinyl tube into a plastic cup in the center of the rear wall opposite the touchscreen. Illumination during experimental sessions was provided by a house light mounted on the upper rear wall of the chamber. Both the pellet dispenser and the house light were controlled by a serial I/O interface. A separate computer controlled each chamber, using programs developed in MATLAB^®^ with Psychtoolbox-3 extensions (Brainard, 1997; Pelli, 1997) (http://psychtoolbox.org/).

#### 2.2.3 Stimuli

The same set of 512 videos used with human subjects was also used with the pigeons. Training and testing sets were created in advance by sorting the videos from each category into 10 equally populated quantiles, based on the EF range for the category. We then randomly selected five videos per bin to create the testing set, for a total of 100 videos (50 per category). The remaining 412 videos (206 per category) constituted the training set. This sampling process was done on a subject-by-subject basis. An alternative set of the training videos in which the opacity of the segmentation ranged from 100 to 0% (in steps of 10%) was also used (see Figure 1C). A white star served as the start stimulus, and a yellow (RGB: 255, 255, 0) and a blue (RGB: 0, 0, 255) rectangle served as choice buttons. Lastly, the segmented video with the median EF relative to the EchoNet-Dynamic dataset was used for pre-training.

#### 2.2.4 Procedure

##### 2.2.4.1 Pre-training

Prior to starting the experiment, pigeons underwent a single pre-training session consisting of 100 trials. See Figure 2B for an overview of a typical trial. Each trial started with the display of a start stimulus. Once pecked, the start stimulus disappeared and the pre-training video was displayed in the center of the screen. The first peck made to the video after 7 s caused two choice buttons to appear to the left and right sides of the video. A final peck to either of the choice buttons terminated the trial. During pre-training, trials were non-differentially reinforced, so no matter which button was chosen, one to three food pellets were randomly delivered and a 6 to 7 s intertrial interval (ITI) ensued.

##### 2.2.4.2 Training with segmented videos

Pigeons received daily training sessions until their proportion of correct responses in a session was 0.70 or more for both categories. In contrast to humans, pigeons could be trained extensively with repeating stimuli, so we required this higher criterion to ensure robust learning. In each session, 100 segmented videos (50 per category) from the training set were randomly presented. The trial structure for training was identical to pre-training, except for the observing requirement and the presence of corrective feedback. The observing requirement for each video started at 7 s and increased across sessions based on each pigeon's level of performance; final values ranged from 15 to 20 s depending on the pigeon. If pigeons chose the button corresponding to the category of the video being displayed, then one to three food pellets were randomly delivered; otherwise, no food was delivered and a correction trial with the same video was given after the ITI (6 to 7 s), until they made the correct choice. Data from correction trials were excluded from all statistical analyses. The button assignment for each category was counterbalanced across birds, such that the blue and yellow buttons were associated with normal and abnormal videos, respectively, for two of the birds, and vice versa for the other two birds.

##### 2.2.4.3 Testing with novel segmented videos

Unlike the humans, who were trained with non-repeating stimuli, the pigeons were trained with stimuli that could be repeated. To assess whether the pigeons had formed category knowledge beyond mere memorization, pigeons completed 20 daily sessions containing a random mixture of 100 segmented training trials and 20 trials with segmented videos from the testing set. The trial structure was identical to training, except that testing trials involved non-differential reinforcement.

##### 2.2.4.4 Testing with familiar non-segmented videos

Pigeons completed 20 daily sessions containing 100 segmented training trials and 20 testing trials with non-segmented videos from the training set. Testing with familiar but non-segmented videos allowed us to assess whether pigeons were at all capable of generalizing to that video modality before testing them with novel non-segmented videos. Again, the trial structure was identical to training, except that testing trials involved non-differential reinforcement.

##### 2.2.4.5 Opacity reduction phase

Prior work has shown that fading—the progressive removal of a salient, assistive cue—can aid in the learning of multidimensional categories (Pashler and Mozer, 2013). To aid pigeons in learning to categorize non-segmented videos, we employed a fading procedure in which we gradually reduced the opacity of the segmentation in the training videos. Starting with videos in which the segmentation was at 100% opacity, pigeons received daily sessions containing 100 training trials at a set opacity (see Figure 1C). Once pigeons achieved a proportion of correct responses of 0.70 for 3 days in a row, the opacity of the segmentation was reduced by 10%. Pigeons that met this criterion for videos at 0% opacity (i.e., fully non-segmented videos) were to be tested with novel non-segmented videos to assess generalization. We did not specify an alternative terminating criterion; instead, we terminated data collection when we determined, via visual inspection, that performance had plateaued for all pigeons.

#### 2.2.5 Analyses

##### 2.2.5.1 Overview

All pigeon analyses were conducted in the same manner as the human analyses, including model selection and model type. The original data and code used for the analyses are available in the OSF repository at https://doi.org/10.17605/OSF.IO/7C9HK.

##### 2.2.5.2 Assessing acquisition with segmented videos

All the pigeons met the training criterion, so there was no need to exclude those that did not. However, pigeons met criterion at different rates; from fastest to slowest, they met criterion on sessions 42, 60, 61, and 90. To focus on initial learning and ensure equal data contribution from each subject, we limited our analysis to the first 42 sessions: the minimum required for any pigeon to meet criterion. Like with the humans, we accounted for non-linear learning rates and potential differences in performance across categories by including the logarithm of 700-trial blocks (1–6, centered on the last block) and category (contrast coded as before) as fixed factors. The model also included random subject intercepts and random subject slopes for category and the interaction between category and block.

##### 2.2.5.3 Assessing generalization to novel segmented videos

To evaluate the pigeons' ability to generalize their learning to novel segmented videos, we compared their performance on trials with novel segmented videos to their performance on trials with segmented training videos during the same phase. Due to an experimenter error, one pigeon received 19 testing sessions instead of 20. For purposes of analysis, the data for this bird was thus limited to its first 19 sessions, while the data for the other birds included all 20 sessions. The model used to assess these data included video type (contrast coded with segmented training at 0.5 and novel segmented testing at −0.5) and category (contrast coded as before) as fixed factors. The model also included random subject intercepts and random subject slopes for category.

##### 2.2.5.4 Assessing generalization to familiar non-segmented videos

To assess the pigeons' ability to generalize to familiar non-segmented videos, we compared their performance on trials with familiar non-segmented videos to their performance on trials with segmented training videos during the same phase. The model used to assess these data included video type (contrast coded with segmented training at 0.5 and familiar non-segmented testing at −0.5) and category (contrast coded as before) as fixed factors. The model also included random subject intercepts.

##### 2.2.5.5 Assessing the opacity reduction phase

Recall that the pigeons were required to maintain a proportion of correct responses of at least 0.70 for 3 consecutive days before progressing to a reduced opacity. Our original intention was to assess pigeons' generalization to novel non-segmented videos after they met criterion at an opacity of 0% (i.e., after they met criterion with fully non-segmented videos). Unfortunately, because no bird met criterion at 0% opacity, we were unable to assess this. Instead, we present graphs depicting their performance during this phase up until we terminated data collection.

## 3 Results

### 3.1 Humans

#### 3.1.1 Acquisition with segmented vs. non-segmented videos

The categorization task proved to be difficult for humans, with only 28 out of 62 subjects from the Segmented Training Group and 16 out of 63 subjects from the Non-segmented Training Group meeting criterion in the last block of training. The z-test revealed that significantly more subjects from the Segmented Training Group met criterion than those in the Non-segmented Training Group (*p* = 0.033).

**Figure 3:**
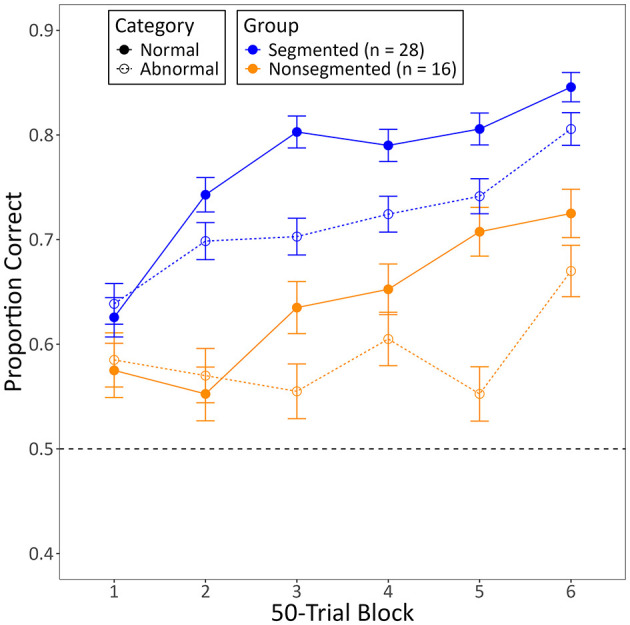
The mean proportion of correct responses made by human subjects to each category during training with either segmented or non-segmented videos. Only subjects who met the training criterion are included. Error bars represent the within-subject standard error of the mean (Morey, 2008), and the dotted line indicates chance-level performance (0.50).

Figure 3 shows the mean proportion of correct responses these subjects made for each category as a function of 50-trial blocks during training with segmented (*n* = 28) or non-segmented videos (*n* = 16). The model used to assess these data revealed a reliable increase in categorization accuracy across blocks of training for both groups (*b* = 0.42, 95% CI [0.31, 0.53], *Z* = 7.34, *p* < 0.001). This increase was faster for normal videos than for abnormal videos (*b* = 0.30, 95% CI [0.17, 0.43], *Z* = 4.64, *p* < 0.001), with subjects also being more accurate in categorizing normal videos than abnormal videos by the end of training (*b* = 0.52, 95% CI [0.33, 0.70], *Z* = 5.39, *p* < 0.001). More importantly, this increase was also faster for subjects trained with segmented videos than for those trained with non-segmented videos (*b* = 0.31, 95% CI [0.08, 0.53], *Z* = 2.68, *p* = 0.007), with those trained with segmented videos also being more accurate than those trained with non-segmented videos by the end of training (*b* = 0.91, 95% CI [0.50, 1.31], *Z* = 4.42, *p* < 0.001). The model did not disclose any other significant interactions, either between category and group (*b* = 0.10, 95% CI [−0.27, 0.48], *Z* = 0.53, *p* = 0.596) or among all three variables (*b* = −0.03, 95% CI [−0.28, 0.23], *Z* = −0.22, *p* = 0.827).

#### 3.1.2 Generalization from segmented to non-segmented videos

**Figure 4:**
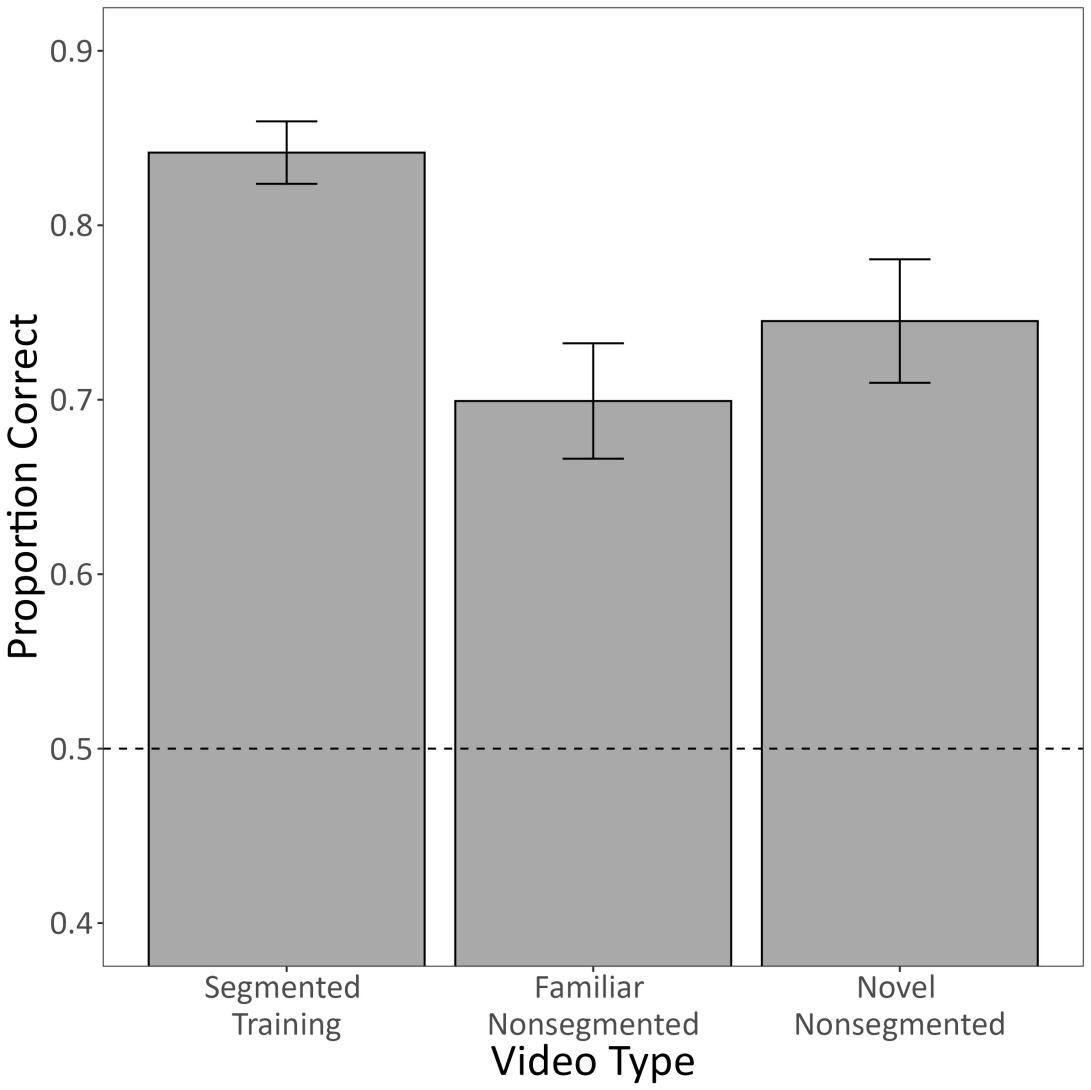
The mean proportion of correct responses made by human subjects from the Segmented Training Group who were tested with non-segmented videos. Only subjects who met the training criterion are included (*n* = 12). Segmented training videos constitute segmented videos from the last 50-trial block of training. Familiar non-segmented videos constitute videos from testing that were non-segmented versions of training videos. Novel non-segmented videos constitute non-segmented videos from testing that were completely novel. Error bars represent the within-subject standard error of the mean (Morey, 2008), and the dotted line indicates chance-level performance (0.50). See Supplementary material 3 for category-level accuracies.

Of the 28 subjects that met criterion during segmented training, 12 were in the group that was also tested with non-segmented videos. Figure 4 shows the mean proportion of correct responses that these subjects (*n* = 12) made to segmented videos in the last block of training, familiar non-segmented testing videos, and novel non-segmented testing videos. Unsurprisingly, the model used to assess these data revealed accuracy to be significantly lower for both familiar non-segmented videos (*b* = −0.84, 95% CI [−1.19, −0.49], *Z* = −4.75, *p* < 0.001) and novel non-segmented videos (*b* = −0.67, 95% CI [−1.07, −0.28], *Z* = −3.34, *p* < 0.001) compared to the segmented training videos. More importantly, humans categorized both familiar and novel non-segmented videos at above chance levels (both *p* < 0.001).

#### 3.1.3 Generalization from non-segmented to segmented videos

**Figure 5:**
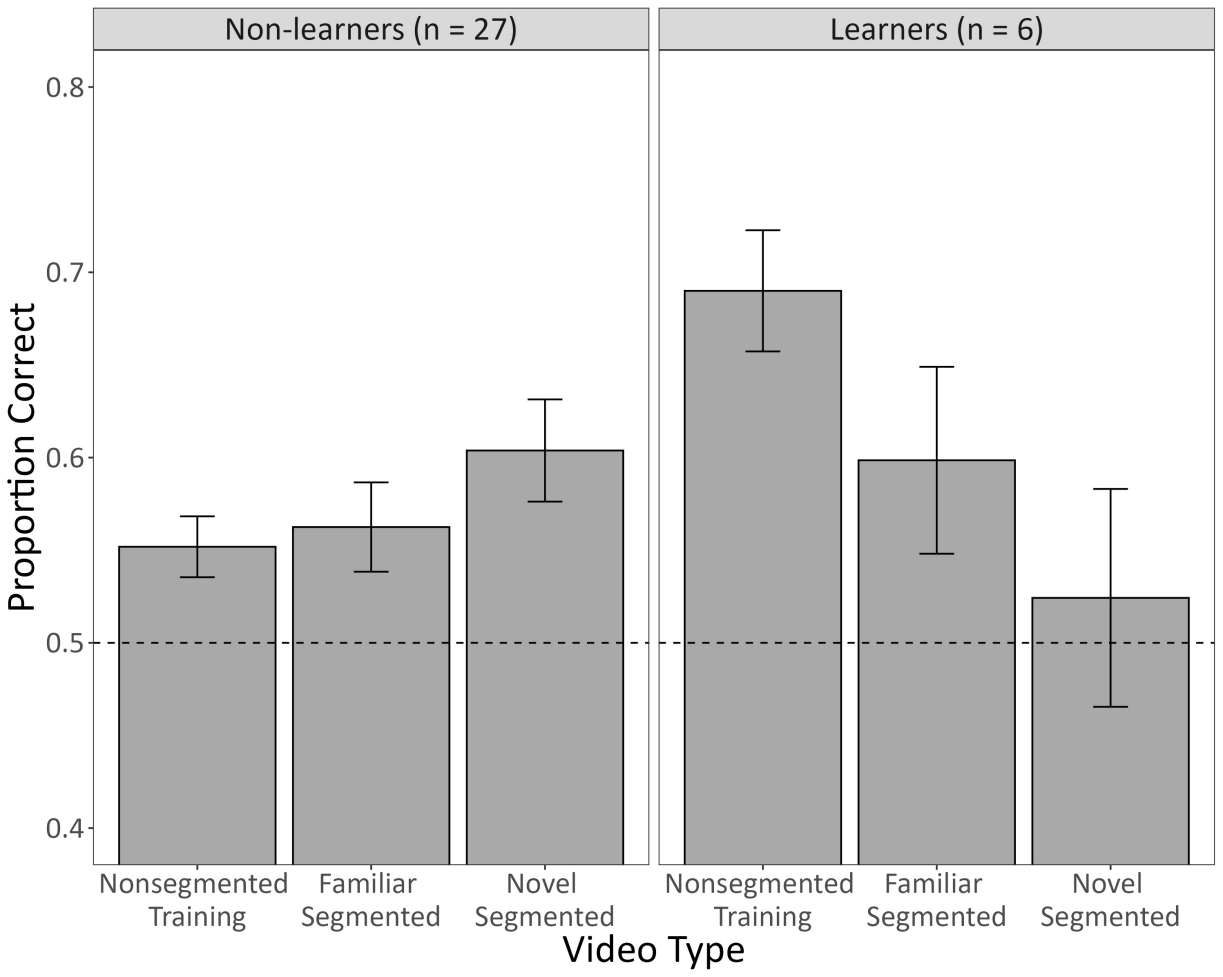
The mean proportion of correct responses made by human subjects from the Non-segmented Training Group who were tested with segmented videos (*n* = 33). Non-learners (*n* = 27) are those that failed to meet the training criterion, whereas learners (*n* = 6) are those that met the criterion. Non-segmented training videos constitute non-segmented videos from the last 50-trial block of training. Familiar segmented videos constitute videos from testing that were segmented versions of training videos. Novel segmented videos constitute segmented videos from testing that were completely novel. Error bars represent the within-subject standard error of the mean (Morey, 2008), and the dotted line indicates chance-level performance (0.50). See Supplementary material 4 for category-level accuracies.

Figure 5 shows the mean proportion of correct responses that subjects (*n* = 33) made to non-segmented videos in the last block of training, familiar segmented testing videos, and novel segmented testing videos as a function of whether subjects achieved criterion (*n* = 6) or not (*n* = 27).

The model used to assess these data revealed that subjects who met the training criterion generally outperformed subjects who did not (*b* = 0.60, 95% CI [0.27, 0.93], Z = 3.56, *p* < 0.001). Interestingly, the model also revealed a significant interaction between the novel segmented testing video contrast and learner (*b* = −0.94, 95% CI [−1.45, −0.43], Z = −3.63, *p* < 0.001). Follow-up analyses indicated that subjects who met the training criterion experienced a generalization decrement to these novel videos (*p* = 0.002), whereas those who did not meet the criterion actually performed better with these novel videos (*p* = 0.040). All other effects assessed in the main model were not significant, including the familiar segmented testing video contrast (*b* = −0.18, 95% CI [−0.42, 0.05], Z = −1.54, *p* = 0.123), the novel segmented testing video contrast (*b* = −0.25, 95% CI [−0.50, 0.01], Z = −1.89, *p* = 0.058), and the interaction between the familiar segmented testing video contrast and learner (*b* = −0.44, 95% CI [−0.90, 0.03], Z = −1.85, *p* = 0.065).

### 3.2 Pigeons

#### 3.2.1 Acquisition with segmented videos

**Figure 6:**
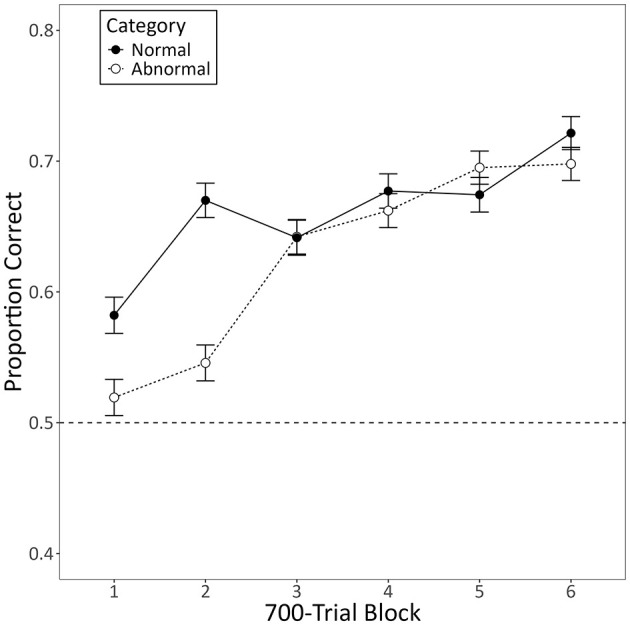
The mean proportion of correct responses made by pigeons to each category during training with segmented videos. The data is limited to the minimum number of daily sessions required for any pigeon to meet the training criterion (i.e., 42 sessions). Error bars represent the within-subject standard error of the mean (Morey, 2008), and the dotted line indicates chance-level performance (0.50).

All of the pigeons met the training criterion. Figure 6 shows the mean proportion of correct responses the pigeons made for each category during the first 42 training sessions as a function of 700-trial blocks. The model used to assess these data disclosed a significant increase in categorization accuracy across blocks of training (*b* = 0.39, 95% CI [0.34, 0.44], *Z* = 14.45, *p* < 0.001). Contrary to the humans, though, neither category was learned significantly faster or slower than the other (*b* = −0.23, 95% CI [−0.52, 0.06], Z = −1.58, *p* = 0.115), and there was no reliable difference in accuracy between the categories by the end of training (*b* = −0.04, 95% CI [-0.75, 0.67], Z = −0.11, *p* = 0.915).

#### 3.2.2 Generalization to novel segmented videos

**Figure 7:**
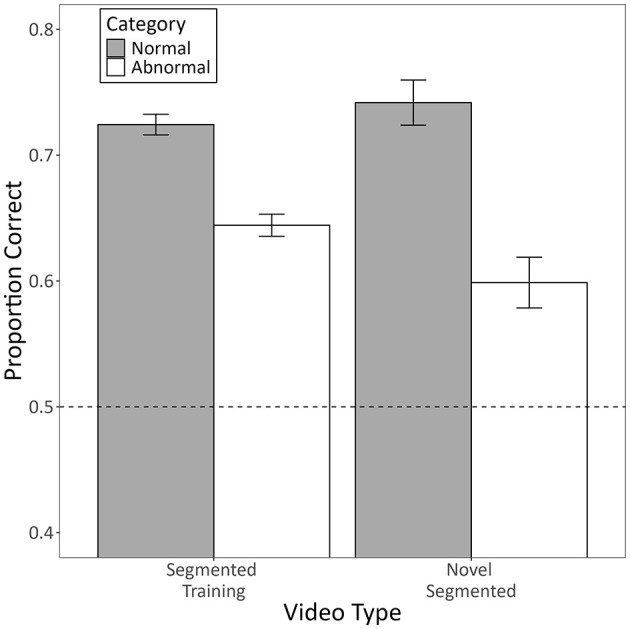
The mean proportion of correct responses made by pigeons to each category during testing with novel segmented videos. Segmented training videos constitute familiar videos from training that were presented in the same phase as the novel testing videos. Novel segmented videos constitute completely novel testing videos presented in the same phase as the training videos. Error bars represent the within-subject standard error of the mean (Morey, 2008), and the dotted line indicates chance-level performance.

Figure 7 shows the mean proportion of correct responses the pigeons made during testing with novel segmented videos as a function of video type (segmented training or novel segmented) and category (normal or abnormal).

The model revealed a significant interaction between category and video type (*b* = −0.29, 95% CI [−0.52, −0.05], *Z* = −2.39, *p* = 0.017). Follow-up contrasts indicated that accuracy was lower for novel segmented videos compared to segmented training videos, but only for videos from the abnormal category (*p* = 0.015). Videos from the normal category exhibited no such difference (*p* = 0.310). The interaction was qualified by a significant main effect of category, such that pigeons were generally more accurate in categorizing normal videos than abnormal videos (*b* = 0.54, 95% CI [0.01, 1.07], *Z* = 2.00, *p* = 0.046), but there was no main effect of video type (*b* = 0.05, 95% CI [−0.06, 0.17], Z = 0.88, *p* = 0.379). Most importantly, pigeons categorized novel segmented videos from both categories at above chance levels (both *ps* < 0.001).

#### 3.2.3 Generalization to familiar non-segmented videos

**Figure 8:**
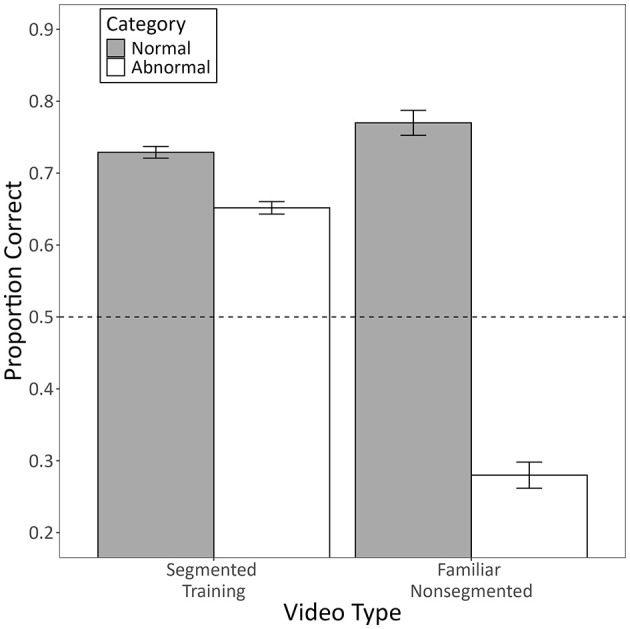
The mean proportion of correct responses made by pigeons to each category during testing with familiar non-segmented videos. Segmented training videos constitute familiar videos from training that were presented in the same phase as the non-segmented testing videos. Familiar non-segmented videos constitute testing videos that were non-segmented versions of training videos, presented in the same phase as the training videos. Error bars represent the within-subject standard error of the mean (Morey, 2008), and the dotted line indicates chance-level performance.

Figure 8 shows the mean proportion of correct responses the pigeons made during testing with familiar non-segmented videos as a function of video type (segmented training or familiar non-segmented) and category (normal or abnormal).

The model used to assess these data revealed a significant interaction between category and video type (*b* = −1.79, 95% CI [-2.04, −1.55], *Z* = −14.32, *p* < 0.001). Follow-up contrasts indicated that, for videos from the abnormal category, accuracy for non-segmented videos was significantly lower than that for segmented videos (*p* < 0.001). This effect was so dramatic that performance on these non-segmented videos was significantly below chance (*p* < 0.001; see Figure 8). Conversely, for videos from the normal category, accuracy for non-segmented videos was significantly *higher* than that for segmented videos (*p* = 0.017). These results strongly suggest that the pigeons merely exhibited a bias on trials with non-segmented videos, such that they were more likely to choose the report button corresponding to the normal category than the button corresponding to the abnormal category. This effect was qualified by pigeons' significantly higher accuracy for videos from the normal category compared to the abnormal category (*b* = 1.26, 95% CI [1.14, 1.38], *Z* = 20.13, *p* < 0.001), as well as their significantly higher accuracy for segmented videos compared to non-segmented videos (*b* = 0.68, 95% CI [0.55, 0.80], *Z* = 10.82, *p* < 0.001).

#### 3.2.4 Opacity reduction phase

**Figure 9:**
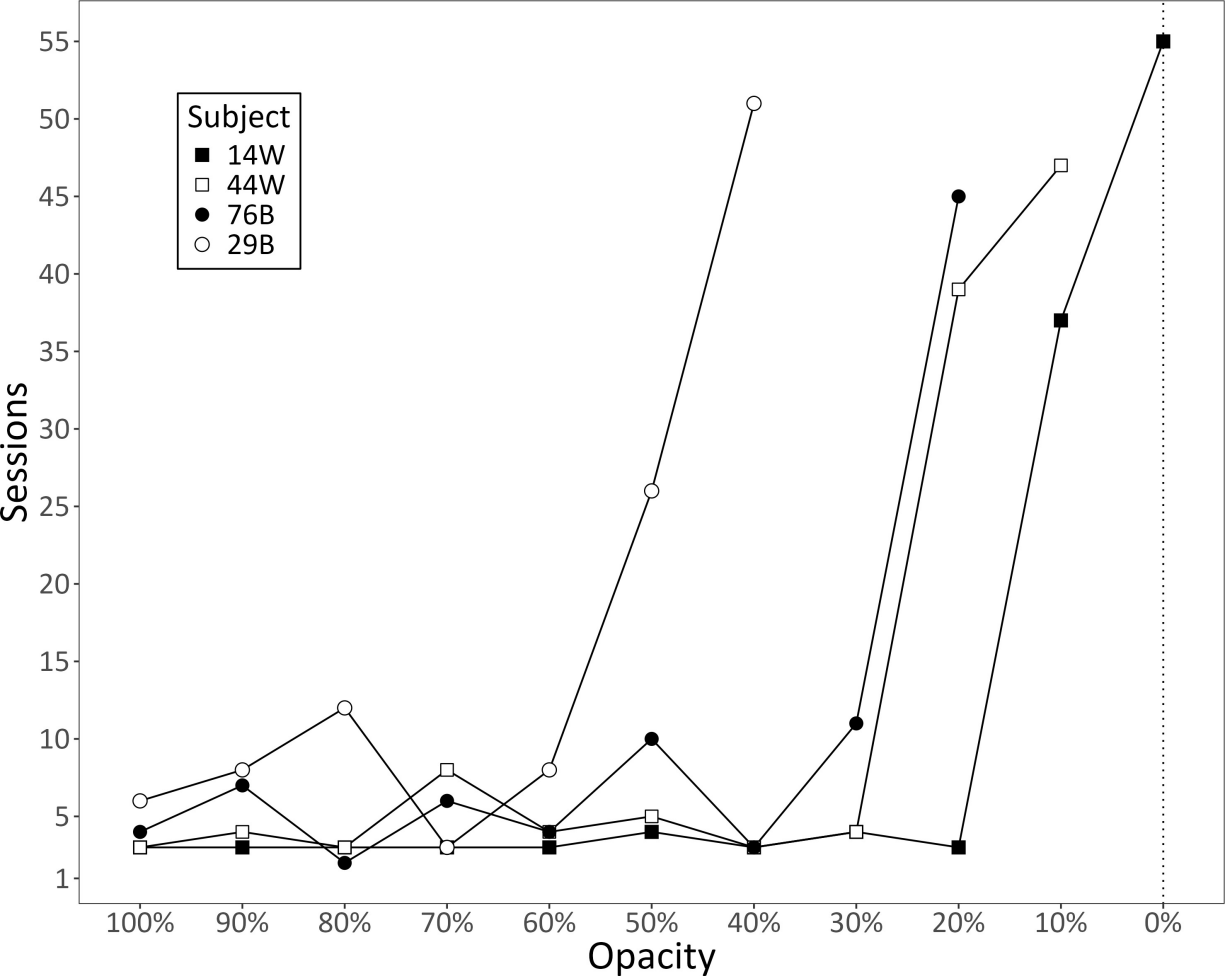
The number of daily sessions pigeons spent at each opacity level. The last point for each bird represents the number of sessions they received at their last opacity level before we terminated data collection. Each point before that represents the number of sessions that each bird took to meet criterion for each opacity. The dotted line indicates the opacity the pigeons were required to meet criterion for to be tested with novel non-segmented videos; no pigeon met criterion at this opacity.

**Figure 10:**
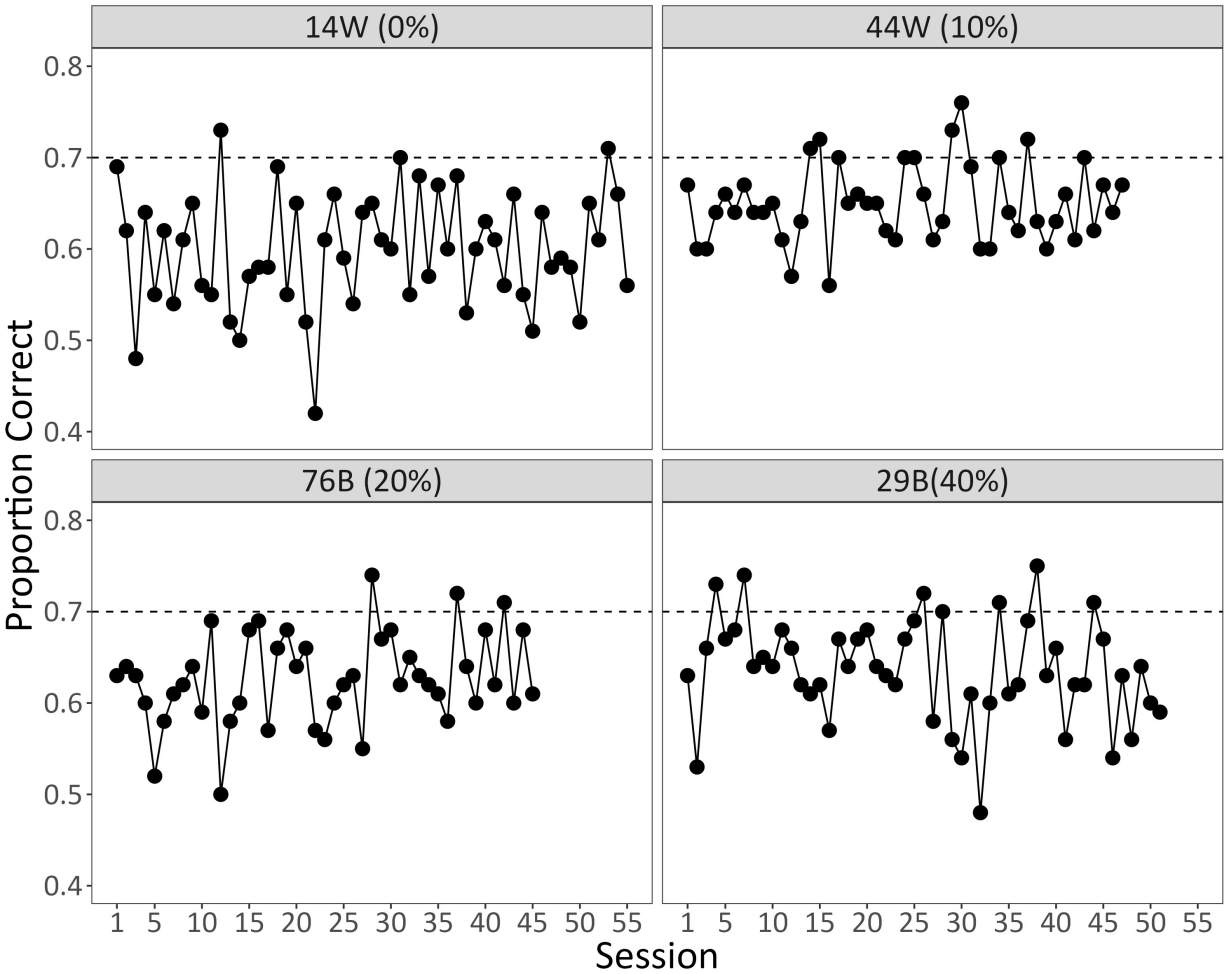
The daily proportion of correct responses each pigeon made during the last opacity they were exposed to. The last opacity for each bird is displayed in parentheses. The dotted line indicates the level of performance (0.70) that pigeons needed to maintain for at least 3 days to progress.

Figure 9 shows the number of days the pigeons spent at each opacity. Although they initially progressed at a relatively steady rate, each bird eventually hit a point at which they struggled to meet the criterion. Only one of the birds ever progressed to an opacity of 0%, but even it failed to meet the criterion for these videos. Figure 10 shows pigeons' accuracy during each day of training at the last opacity they were exposed to. Given the stagnant levels of performance after extended training, it seemed reasonable to terminate data collection when we did. Thus, our fading procedure did not support pigeons' generalization to non-segmented videos.

## 4 Discussion

We evaluated the efficacy of EchoNet-Dynamic as a technological aid for humans and pigeons diagnosing cardiac function. Specifically, we asked: (1) Does EchoNet-Dynamic's segmentation enhance human learning? (2) Does adding the segmentation benefit people previously trained with non-segmented videos? (3) Can pigeons learn with segmented videos? (4) Does segmented training support generalization to non-segmented videos in both species?

We found that more humans trained with segmented videos successfully learned the task than humans trained with non-segmented videos. They also learned more quickly and exhibited higher diagnostic accuracy by the end of training (Figure 3). Despite this, there was no general benefit to adding the segmentation after non-segmented training. Instead, those that had successfully learned with non-segmented videos performed worse with segmented videos, whereas those that hadn't successfully learned performed better, but only if the segmented videos were completely novel (Figure 5). Pigeons, like humans, could learn to categorize segmented videos (Figures 6, 7). However, only humans could generalize their learning to non-segmented videos (Figures 4, 8). Even when given a training regime meant to wean them off EchoNet-Dynamic's segmentation, no pigeon could achieve accurate generalization to non-segmented videos (Figures 9, 10). Our results provide initial evidence of the segmentation's utility as a visual aid: it enhances learning outcomes for humans and provides a means of learning what might otherwise be a difficult task for pigeons. Despite these promising results, further work is needed to fully understand how and when the segmentation can benefit each species.

Firstly, it is unclear why adding the segmentation yielded no general improvement after non-segmented training. One possibility is that the segmentation masked the features to which people had attended during non-segmented training. This seems counterintuitive given that people who underwent segmented training could still accurately categorize non-segmented videos. Moreover, this explanation fails to account for why subjects who initially failed to learn with non-segmented videos only benefitted from the segmentation when it was applied to completely novel videos. These results suggest there is more to EchoNet-Dynamic's segmentation than a mere enhancement of the features relevant to diagnosis. Future research should address this by including post-experiment interviews, which we did not include in our study. This could help reveal strategies or cues used by participants. Future work should also systematically manipulate the segmentation itself. This could help distinguish between whether the segmentation operates via low-level salience or higher-level attentional guidance. Evaluating what features of the segmentation most effectively improve performance and how is essential to successfully implementing it as a visual aid.

In addition to better understanding how and when segmentation is beneficial, it is also important to assess how other stimulus properties impact performance. In the present study, for example, the abnormal category encompassed a wider EF range than the normal category due to unaccounted for constraints in the EF distribution of the available video set (see Figure 1B): videos with lower EFs were more infrequent and covered a wider EF range than videos with higher EFs. This increased within-category variability likely reduced the cohesiveness of videos from the abnormal category, which could explain why human subjects learned this category more slowly. Future research should manipulate EF range and other stimulus characteristics to understand what is most conducive to learning and generalization for these stimuli. As with research on the properties of the segmentation itself, this can help inform enhanced training procedures.

Another potential future direction is to explore how task instructions affect performance. In our own study, we did not instruct human subjects on what features were relevant for diagnosis. This is one possible explanation for why so few subjects learned the task: our subjects were naïve non-clinicians, so they could only learn via trial-and-error. If the goal is to eventually use the segmentation to aid learning in a clinical context, then providing subjects with a basic understanding of what features they should attend to is necessary. This would better reflect the level of understanding that novice clinicians might have. If the primary goal is to instead make comparisons across species, then it may be more beneficial to ensure that task constraints like this are as similar as possible across species. Either way, future work should be mindful of what knowledge is afforded to subjects before beginning the task.

Before discussing why pigeons differed from humans in their ability to generalize from segmented to non-segmented videos, there are several procedural differences in our study that warrant consideration. Pigeons received substantially more training than humans. Their training videos were also repeatable, whereas they were non-repeatable for humans. Furthermore, testing trials were intermixed with training trials for pigeons but occurred separately for humans. Lastly, their observation requirements differed: pigeons were required to peck the videos for the response keys to appear, while humans only needed to view each video for at least 2s. Future work aiming to make cross-species comparisons should control for these differences; otherwise, apparent species differences may reflect procedural confounds.

One of the purposes of our study was to evaluate whether pigeons' utility as a low-cost, high-throughput model for medical classification extends to a domain where motion is diagnostic. Although pigeons learned with EchoNet-Dynamic's segmentation, they failed to generalize to non-segmented videos, unlike humans. Differences in visual processing, learning, memory, and procedural factors may underlie this disparity. Our study was not designed to distinguish among these possibilities, but prior work shows that in the presence of multiple cues, pigeons preferentially attend to those involving color (Jones, 1954; Lazareva et al., 2005; Navarro et al., 2020). Additionally, pigeons experience attentional tradeoffs when attending to multiple cues—tradeoffs that can be exacerbated in particularly challenging tasks (Gottselig et al., 2001). Thus, pigeons likely relied heavily on EchoNet-Dynamic's segmentation during training, testing, and during the Opacity Reduction Phase. If so, our results provide limited support for using pigeons as surrogates for studying the medical classification of these stimuli. Because their learning depended entirely on the segmentation, they likely did not form a medical concept comparable to humans. Future research should test pigeons' ability to learn with non-segmented videos and develop training procedures that minimize reliance on segmentation. This would clarify whether pigeons can model human performance within this challenging medical domain.

## Data Availability

The data and code for this study is available in the OSF repository at https://doi.org/10.17605/OSF.IO/7C9HK.
