# Supplementary material for: Categorization of echocardiograms by humans and pigeons
Source: Front Psychol. 2026 Jan 9;16:1680346. doi: 10.3389/fpsyg.2025.1680346 (PMC12827666; doi:10.3389/fpsyg.2025.1680346)
Supplement: Supplementary file 1 [file Data_Sheet_1.docx]

Supplementary Material

# Instructions Given to Human Participants

Thanks for participating in this study!

In this study, you will be asked to play the role of a medical student learning to diagnose echocardiograms. On each trial, you will be shown a video depicting a beating heart. Your task is to decide whether this heart is normal or dysfunctional, by pressing keys on your keyboard. You will be able to make a response after 2 seconds (indicated by a prompt appearing under the video). Press <F, J> if you think the heart is normal. Press <J, F> if you think the heart is dysfunctional.

In order to help you learn to diagnose these videos, you will receive feedback after each of your choices. You will have to diagnose many videos, and you can take as long as you like to diagnose them (your reaction time is not important in this study), but we recommend that you don’t spend too long on any one of the videos. Typically, this experiment will take around 30 minutes to complete. Please don't write anything down during the experiment.

# Quiz Given to Human Participants After Task Instructions

*Note*: Correct answers are in bold

- Question 1: What will you be doing during this task?
  - I will complete a questionnaire about my personality.
  - On each trial I will see a circle, and will be asked what color it is.
  - **I will be playing the role of a medical student, and will learn to diagnose heart videos as normal/dysfunctional.**
- Question 2: How often will you receive feedback on your decisions?
  - Never.
  - **After every choice.**
  - After every 10 choices.
- Question 3: How will you report your decisions?
  - **By pressing keys on my keyboard.**
  - By speaking into my microphone.
  - By clicking with my mouse.

# Category-level Accuracies During Human Generalization to Nonsegmented Videos


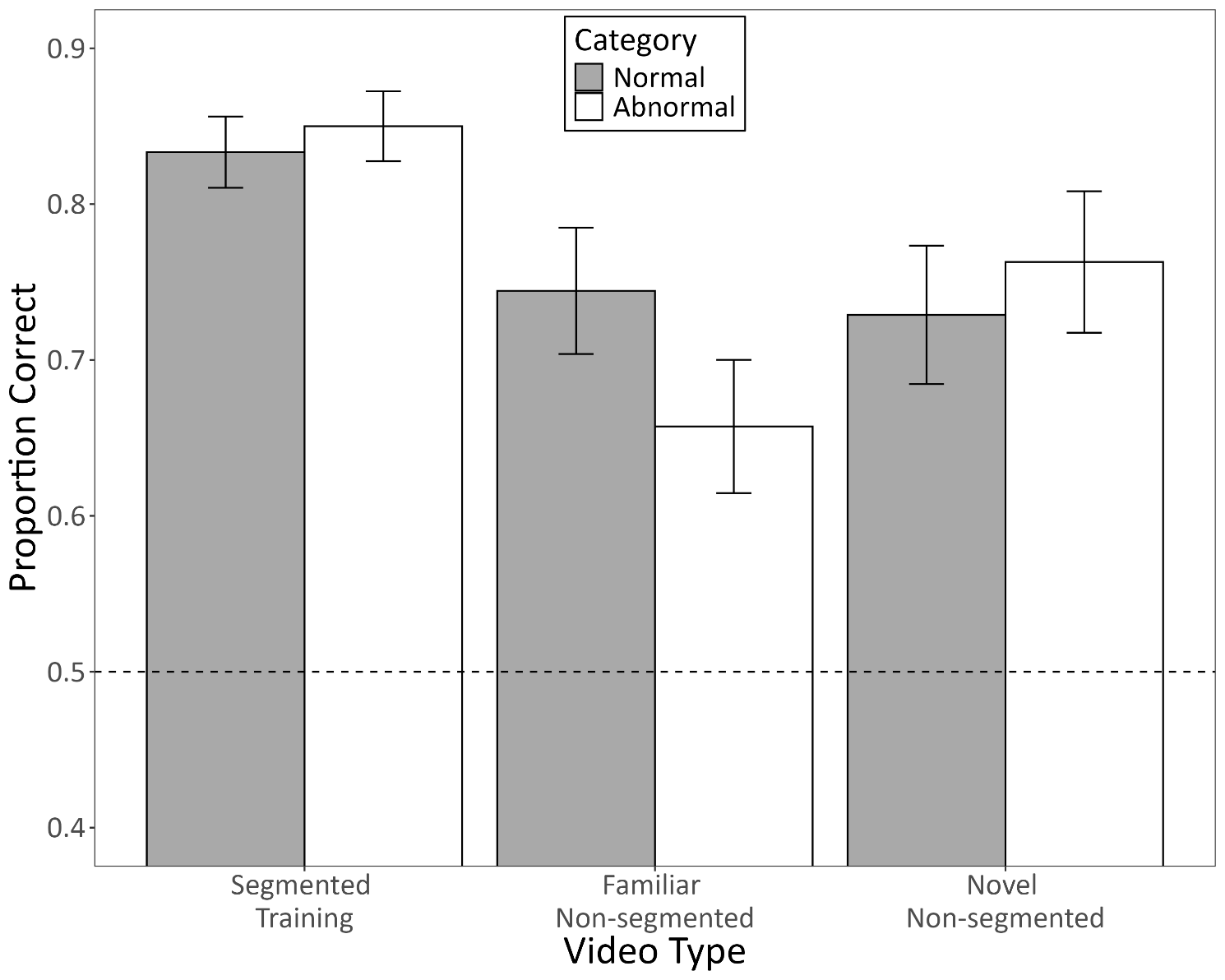


*Note*. The data presented in this figure is the same as that presented in Figure 4 of the main text, except that the mean proportion of correct responses is also calculated as a function of whether videos were from the normal or abnormal category. Only subjects in the Segmented Training Group who were tested and who met the training criterion are included (*n* = 12). Segmented training videos only include videos presented in the last 50-trial block of training. Error bars represent the within-subject standard error of the mean (Morey, 2008), and the dotted line indicates chance-level performance (.50).

# Category-level Accuracies During Human Generalization to Segmented Videos


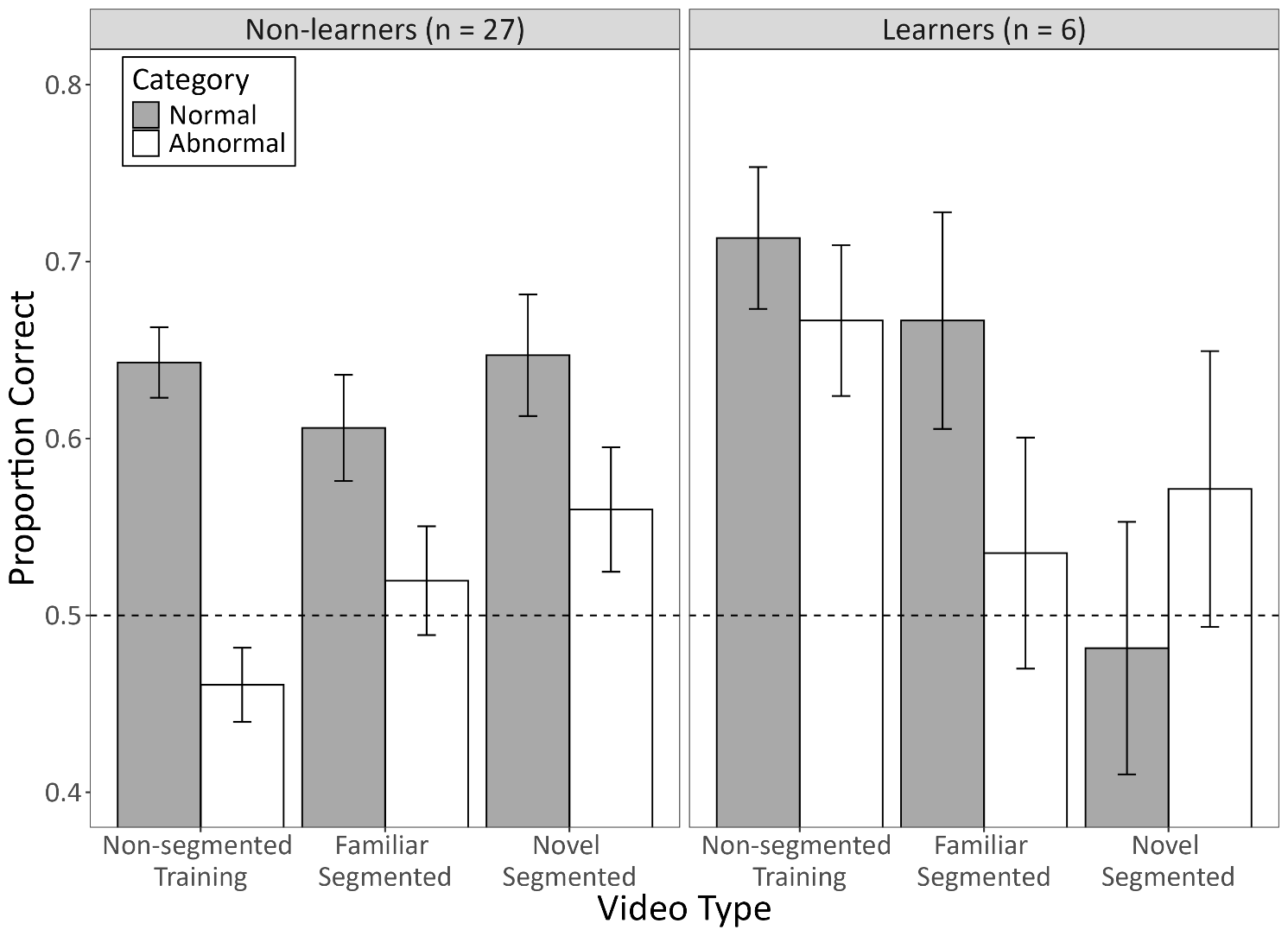


*Note*. The data presented in this figure is the same as that presented in Figure 5 of the main text, except that the mean proportion of correct responses is also calculated as a function of whether videos were from the normal or abnormal category. Only subjects in the Nonsegmented Training Group who were tested (*n*  = 33) are included. Non-learners are those that failed to meet the training criterion, whereas learners are those that met the criterion. Nonsegmented training videos only include videos presented in the last 50-trial block of training. Error bars represent the within-subject standard error of the mean (Morey, 2008), and the dotted line indicates chance-level performance (.50).

# References

1. Morey, R. D. (2008). Confidence intervals from normalized data: A correction to Cousineau (2005). *Tutor. Quant. Methods Psychol.* 4(2), 61–64. <https://doi.org/10.20982/tqmp.01.1.p042>
